# Therapeutics of Artificial Serum

**Published:** 1906-10

**Authors:** 


### Therapeutics of Artificial Serum.

Hubert Richardson, Baltimore, (*Dietetic and Hygienic Gazette,* February, 1906,) says the blood plasma contains inorganic salts in certain definite proportions, forming a fluid of definite specific gravity adapted for holding in solution the cellular aliments as well as the effete matters of metabolism which are eliminated by the kidneys. A change in the proportion of these salts produces pathologic conditions and interferes with nutrition and elimination, causing starvation or autointoxication.

Arteriosclerosis is due to deposits of calcium and cholesterine in the arterial walls. Trunecek, reasoning that the normal condition of the plasma could be restored by the administration of the inorganic salts in normal proportion, tried this treatment in arteriosclerosis and obtained excellent results therefrom.

When an excess of any substance is eliminated by the kidneys, the elimination of sodium chloride is correspondingly increased. The proportion of the inorganic salts, which is thus disturbed, can be reestablished by the use of these salts. In autointoxication, the result of the non-elimination of toxins produces in the organism, the process of excretion is greatly aided by artificial serum.

In many cases of malnutrition, the chief cause is the decreased power of the various organs to absorb and distribute nutriment, because the plasma is unable to hold the food substances in solution. In mucous gastritis, a pint of distilled water in which four antisclerosin tablets (which consist of the blood salts in the normal proportion) have been dissolved, taken ½ hour before each meal, will answer the same purpose as washing out the stomach. Improvement is generally very rapid; nutrition becomes better, the complexion clears and the patient experiences a gratifying feeling of well-being.

Liver stimulation is resorted to in almost all constitutional diseases, and it is obvious that a solvent for the substances congested in the liver, both as a means of carrying the food products to the organs and as an eliminant to carry off the excess by the kidneys, is of paramount importance. Artificial serum constitutes the ideal treatment, giving the system an excess of the natural eliminants.

Taken in mineral water, the salts are a most satisfactory adjuvant to other treatment, and in arteriosclerosis I invariably obtained marked improvement or arrest of the disease progress after a few months. Ten antisclerosin tablets are dissolved in a quart of distilled water and taken as a regular beverage. Saline solution has long been used intravenously in Asiatic cholera and other diseases and in loss of blood. But a solution of the inorganic salts in their natural proportion is much preferable; there is probably no toxic dose for it when 3 c.c. per minute are intravenously injected.

In the large majority of cases, however, a high enema of artificial serum gives the same result and is easier to administer. Ten antisclerosin tablets in a pint of water make normal artificial serum. Its rectal injection is especially indicated in heat collapse, weaknes from the vomiting of alcoholism and pregnancy, and the like. It acts by raising blood pressure and restores to the blood the volume of fluid lost by hemorrhages or serous effusion, as in diarrhea. It minimizes the danger of post-operative collapse and of shock. In infections and intoxications it aids oxidation and dilution of the toxins, acting as a sort of lavage to the blood; it renews and invigorates the process of phagocytosis which has been impaired by the hypertoxicity of the blood, as has been shown by Sahli. Furthermore, the injection has an undoubted tonic effect upon the nervous system, especially when continued for some time. Among the insane I have had many opportunities of observing the beneficial effect of high rectal enemata of artificial serum. The effects are a more regular heart beat, increased tension, diuresis, increased toxicity of the urine, improved digestion and appetite, and deeper and more regular respiration.

